# Relationships of Cetacea (Artiodactyla) Among Mammals: Increased Taxon Sampling Alters Interpretations of Key Fossils and Character Evolution

**DOI:** 10.1371/journal.pone.0007062

**Published:** 2009-09-23

**Authors:** Michelle Spaulding, Maureen A. O'Leary, John Gatesy

**Affiliations:** 1 Department of Earth & Environmental Sciences, Columbia University, New York, New York, United States of America; 2 Division of Paleontology, American Museum of Natural History, New York, New York, United States of America; 3 Department of Anatomical Sciences, Stony Brook University, Stony Brook, New York, United States of America; 4 Department of Biology, University of California Riverside, Riverside, California, United States of America; Raymond M. Alf Museum of Paleontology, United States of America

## Abstract

**Background:**

Integration of diverse data (molecules, fossils) provides the most robust test of the phylogeny of cetaceans. Positioning key fossils is critical for reconstructing the character change from life on land to life in the water.

**Methodology/Principal Findings:**

We reexamine relationships of critical extinct taxa that impact our understanding of the origin of Cetacea. We do this in the context of the largest total evidence analysis of morphological and molecular information for Artiodactyla (661 phenotypic characters and 46,587 molecular characters, coded for 33 extant and 48 extinct taxa). We score morphological data for Carnivoramorpha, †Creodonta, Lipotyphla, and the †raoellid artiodactylan †*Indohyus* and concentrate on determining which fossils are positioned along stem lineages to major artiodactylan crown clades. Shortest trees place Cetacea within Artiodactyla and close to †*Indohyus*, with †Mesonychia outside of Artiodactyla. The relationships of †Mesonychia and †*Indohyus* are highly unstable, however - in trees only two steps longer than minimum length, †Mesonychia falls inside Artiodactyla and displaces †*Indohyus* from a position close to Cetacea. Trees based only on data that fossilize continue to show the classic arrangement of relationships within Artiodactyla with Cetacea grouping outside the clade, a signal incongruent with the molecular data that dominate the total evidence result.

**Conclusions/Significance:**

Integration of new fossil material of †*Indohyus* impacts placement of another extinct clade †Mesonychia, pushing it much farther down the tree. The phylogenetic position of †*Indohyus* suggests that the cetacean stem lineage included herbivorous and carnivorous aquatic species. We also conclude that extinct members of Cetancodonta (whales + hippopotamids) shared a derived ability to hear underwater sounds, even though several cetancodontans lack a pachyostotic auditory bulla. We revise the taxonomy of living and extinct artiodactylans and propose explicit node and stem-based definitions for the ingroup.

## Introduction

Establishing the position of Cetacea (whales, dolphins and porpoises) within Mammalia has long been a focus of mammalian systematists. The transition from a primitively quadrupedal terrestrial ancestor to a convergently ‘fish-like’ modern mammal species involved changes in numerous character systems. Almost all anatomical systems of living cetaceans are highly modified for an aquatic lifestyle, with dramatic changes seen in areas such as the ear region, skin, limbs, and cranium relative to terrestrial mammals. The study of phylogenetic data that fossilizes (primarily skeletal and dental morphology) has been particularly important because it is by studying extinct species that we can reconstruct the order of character acquisition that led to the origin of Cetacea (see review of studies in [1], [2]).

Continued discovery of fossils that capture transitional stages in cetacean evolution (e.g., [3], [4], [5] ) provides critical new data on how the stem lineage to Cetacea transformed. By incorporating new fossils into increasingly large total evidence (character congruence) analyses, we are beginning to develop a firm understanding of the evolutionary history of this clade and can start testing explicit hypotheses concerning character transformation. For example, ‘Did whales develop ear bones for underwater hearing while still able to easily move on land?,’ or ‘What came first in the whale lineage - dietary change to aquatic carnivory or committed life in water?’ None of these hypotheses can be assessed without a robust test of the sister taxa to the clade Cetacea.

**Figure 1:**
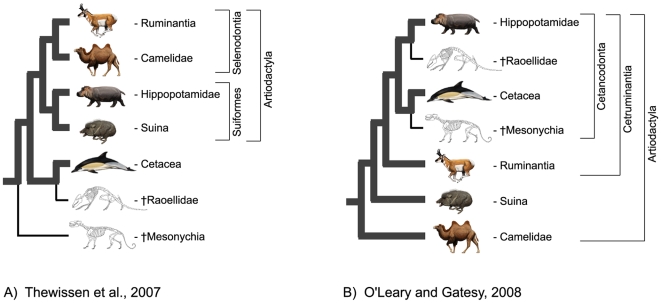
Recent morphological (A) and combined morphological + molecular (B) hypotheses of artiodactylan phylogeny. Most cladistic analyses of morphological characters have supported monophyly of extant terrestrial artiodactylans, traditionally called Artiodactyla, as well as the subclades Suiformes and Selenodontia. Note the variable placements of the enigmatic extinct groups †Raoellidae and †Mesonychia in the different topologies. The deeply nested conflict between phylogenetic hypotheses for Artiodactyla is shown very well by these two recent studies: for the major lineages shown, no clades are shared. Lineages that connect extant taxa in the tree are represented by thick gray branches, and wholly extinct lineages are shown as thin black branches. Illustrations are by C. Buell and L. Betti-Nash.

Subsequent to the last large scale total evidence analyses of the position of cetaceans among mammals [1] new specimens of the extinct †raoellid artiodactylan, †*Indohyus*, were described that potentially offer critical information about the phylogenetic position of Cetacea [3]. Among the new specimens is a skull that preserves quadritubercular dentition (4 major cusps, found in herbivores and omnivores [6], [7]) and a pachyostotic auditory bulla (found in mammals derived for underwater hearing [8]). These features had not previously been recorded in the same individual. Thewissen et al. [3] argued on the basis of these new data that †*Indohyus* was the sister taxon of living and extinct whales. Many aspects of their published phylogenetic tree were, however, highly incongruent with other recent studies (e.g., [1]; see Figure 1), and their phylogenetic results were subsequently challenged [9], [10].

We reevaluate the position of this significant fossil in the context of the largest total evidence analysis of Artiodactyla and relatives to date. We generate 49 new DNA sequences from five nuclear loci and expand our taxonomic sample to include living and extinct Carnivoramorpha (cats, dogs and fossil relatives) and †Creodonta (archaic extinct carnivorous mammals). Carnivoramorpha and †Creodonta may be critical for determining the position of the wholly extinct clade †Mesonychia, which has played a pivotal role in our understanding of the pattern of character evolution in Cetacea (see discussion in [1]). In particular, we are interested to know how the carnivorous (or hypothesized to be carnivorous) taxa (Carnivoramorpha, †Creodonta, †Mesonychia) are related to Cetacea, a highly-specialized carnivorous/piscivorous lineage that is nested within a clade composed primarily of herbivores (Artiodactyla). Inclusion of a variety of taxa such as these, that have dental similarities to early whales, could directly influence tree topology and interpretations of dental evolution on the stem lineage of Cetacea. Because the association of diagnostic †raoellid cranial fossils with postcranial remains [3] has not been convincingly established (noted in [11]), we also examined how exclusion of postcranial information affected the phylogenetic position of †*Indohyus*. To facilitate discussion of key transitional fossils on the stem lineages of living clades, we revise the higher-level taxonomy of Artiodactyla.

Although the focus of this paper is to examine the phylogenetic relationships of †Mesonychia and the †raoellid †*Indohyus*, this study has implications for Ferae (which we recognize as including only Carnivora plus †Creodonta, following [12], [13]; we do not follow the more inclusive Ferae of [14]). The monophyly of both Ferae and †Creodonta has been questioned (e.g., [15], [16]). Despite the long-standing grouping of Ferae [12], this taxon has never been the subject of a rigorous phylogenetic test in a cladistic framework. The modern concept of the †Creodonta, a wholly extinct carnivorous group, includes two sub-clades: †Hyaenodontidae and †Oxyaenidae [15], but the relationships of these taxa needs further testing [16]. The most recent phylogenetic studies of †Creodonta [15], [17] have concentrated on subclades within the group, and did not address the relationships of †Creodonta in a broader framework. Other analyses that included †creodonts (e.g., [13], [18]) have utilized the taxa as outgroups, and did not specifically test the relationship between the two †creodont families. The expansion in taxon sampling of this study not only benefits our understanding of relationships among Cetacea, other artiodactylans, and †Mesonychia, but also those of Ferae and its component clades.

### Revised Taxonomy for Artiodactyla

It has become increasingly important to have phylogenetic names for the ingroup in question, Artiodactyla, to discuss character evolution unambiguously. Other groups have greatly benefited from a revision of taxonomic nomenclature to reflect phylogeny. Previous taxonomies for Artiodactyla have not been based on robust phylogenetic results [14] or have ignored extinct diversity [19]. This has led to confusion in discussions of evolutionary relationships in the clade. This disorder can be rectified, in part, by a new taxonomy that utilizes “crown clades” and “total clades” [20], [21], [22], [23]. A crown grouping is based upon a cluster of extant species, and a total clade is the crown group plus the paraphyletic series of fossils at its base. The core of our new system (Table 1) is a set of traditional, commonly-used taxonomic names that in the past have been applied variously as stem, crown, node, or apomorphy based groups. Furthermore, our taxonomy incorporates information from recent combined phylogenetic studies that have shown a highly consistent set of hierarchical relationships among major clades of Artiodactyla (reviewed in [1]). All higher-level artiodactylan names proposed are being submitted to the Companion Volume to the Phylocode [24]. By formally naming these clades, we hope to assist in providing a unified system of nomenclature for Artiodactyla, consistent with those applied to other mammalian orders.

**Table 1:** Revised Nomenclature of Artiodactyl Taxa.

Artiodactyla	The least inclusive clade that includes *Hippopotamus amphibius*, *Bos taurus*, *Sus scrofa*, and *Camelus dromedaries*
Artiodactylamorpha	Artiodactyla plus all extinct taxa more closely related to extant members of Artiodactyla than to any other living species
Cetacea	The least inclusive clade that includes *Tursiops truncatus* and *Balaena mysticetus*
Cetaceamorpha	Cetacea plus all extinct taxa more closely related to extant members of Cetacea than to any other living species
Hippopotamidae	The least inclusive clade that includes *Hippopotamus amphibius* and *Choeropsis liberiensis*
Hippopotamidamorpha	Hippopotamidae plus all extinct taxa more closely related to extant members of Hippopotamidae than to any other living species
Cetancodonta	The least inclusive clade that includes *Tursiops truncatus* and *Hippopotamus amphibious*
Cetancodontamorpha	Cetancodonta plus all extinct taxa more closely related to extant members of Cetancodonta than to any other living species
Ruminantia	The least inclusive clade that includes *Bos taurus* and *Tragulus napu*
Ruminantiamorpha	Ruminantia plus all extinct taxa more closely related to extant members of Ruminantia than to any other living species
Cetruminantia	The least inclusive clade that includes *Tursiops truncatus* and *Bos Taurus*
Cetruminantiamorpha	Cetruminantia plus all extinct taxa more closely related to extant members of Cetruminantia than to any other living species
Suina	The least inclusive clade that includes *Sus scrofa* and *Tayassu tajacu*
Suinamorpha	Suina plus all extinct taxa more closely related to extant members of Suina than to any other living species
Camelidae	The least inclusive clade that includes *Camelus dromedarius* and *Lama glama*
Camelidamorpha	Camelidae plus all extinct taxa more closely related to extant members of Camelidae than to any other living species

In the new taxonomy (Table 1), we utilize the name Artiodactyla as a crown clade, the monophyletic group that includes the last common ancestor of cattle, antelope, deer, giraffes, musk deer, chevrotains, hippos, pigs, peccaries, and camels, and all of its descendants. Many analyses have supported the nesting of Cetacea several nodes within Artiodactyla (e.g., [25], [26], [27]). This prompted Montgelard et al. [28] to rename the combined group ‘Cetartiodactyla.’ Despite our prior use of the term ‘Cetartiodactyla’ (e.g., [1], [29], [30]), the topological change of placing Cetacea within Artiodactyla was never grounds to retire the name, Artiodactyla, according to rules of phylogenetic nomenclature. ‘Cetartiodactyla’ has gained some traction in the literature, especially among molecular workers, but here we formally retain the name Artiodactyla following the logic entailed in the Phylocode [24]. All groups that we name as crown clades have been robustly supported by combined phylogenetic analyses of molecules and morphology from living and extinct taxa [1], [31] and this study). These include Cetacea, Hippopotamidae, Cetancodonta, Ruminantia, Cetruminantia, Suina, Camelidae, and Artiodactyla (Table 1), which are found in all minimum length trees (even if the strict consensus is sometime unresolved due to unstable fossils).

As suggested by de Queiroz [23], we have applied widely-used artiodactylan names to crown clades. We then added a standard suffix (“-morpha”) to the crown names to identify corresponding total clades. This allows those who are more familiar with extant diversity, presumably the majority of scientists and laypersons, to link extinct diversity broadly to better known extant species. For example, as defined here, Cetacea includes all descendants of the last common ancestor of *Tursiops truncatus* and *Balaena mysticetus* (Table 1). Most biologists are familiar with whales and dolphins, and these species have been referred to as ‘cetaceans’ for a very long time. Assigning the traditional name ‘Cetacea’ to this crown clade informs the user that extinct crown cetaceans are close relatives of whales and dolphins and likely have many of the synapomorphies shared by all extant cetaceans (e.g., obligately-aquatic lifestyle, reduced hindlimbs, tail flukes, flipper-shaped forelimbs, pachyostotic ear bones, etc.). In our new taxonomy, Cetaceamorpha is the total clade defined as Cetacea plus all extinct taxa more closely related to extant cetaceans than to any other living species. This replaces the use of ‘Cetacea’ as a stem clade (sensu [32]). Thus, in addition to the crown clade, Cetaceamorpha includes fossil stem taxa that are successive outgroups to crown Cetacea. Crown and total clade-based taxonomy provides a consistent reference system for both specialists and those less familiar with the systematics of a given clade. As emphasized by [32], a further significant reason to use crown clades and total clades is their unambiguous representation of data directly available for study. Many types of data (e.g., molecular, soft tissue, behavior) are rarely preserved for direct study outside the crown clade, and thus cannot be optimized below the common ancestor of a crown clade ([32], see also Level 1 inference of [33]). Here we also apply no formal taxonomic rank (i.e., Family, Subfamily, etc.) to the names proposed in this paper.

‘Whippomorpha’ was proposed as the name for “Cetacea +Hippopotamidae” [19]. Subsequently, Cetancodonta was offered as a replacement for ‘Whippomorpha’[34]. We support the formalized use of the term “Cetancodonta” for the crown grouping, based upon arguments made by Arnason when the name was first proposed and the problematic nature of using a term ending in -morpha for a crown clade. We formally define Cetancodonta as a node-based taxon, including all species that are descendants of the most recent common ancestor of *Hippopotamus amphibius* and *Tursiops truncatus*. Cetancodontamorpha is applied to the total clade that includes Cetancodonta and all extinct species more closely related to extant cetancodontans than to any other living species (Table 1).

In addition to artiodactylan taxa, we define the completely extinct clade †Mesonychia as a node based taxon that is the common ancestor of †*Hapalodectes leptognathus* and †*Mesonyx obtusidens* and all of its descendants. We have not altered the nomenclature of taxa utilized as outgroups to Artiodactyla in the present study. The sampling for these clades is not comprehensive enough to warrant the re-examination of taxonomic terms in the present study. We should note, however, that Carnivora, as a node-based crown clade, has been defined elsewhere [35], as has the total clade, Carnivoramorpha [13]. Our treatments of Artiodactyla and Cetacea mirror this utilization of a traditional ordinal level name (Carnivora) applied formally to a crown clade. Additional outgroup names used in the discussion below are: crown group Perissodactyla, †Creodonta [13], [15], [35], Ferae [12], [13], [18], and Lipotyphla [12]. For the above terms that have not yet been formally defined cladistically, their current compositions should be unambiguous given the provided references and the taxa in our analysis.

## Results and Discussion

### Minimum Length Trees and Comparisons to Previous Hypotheses

**Figure 2:**
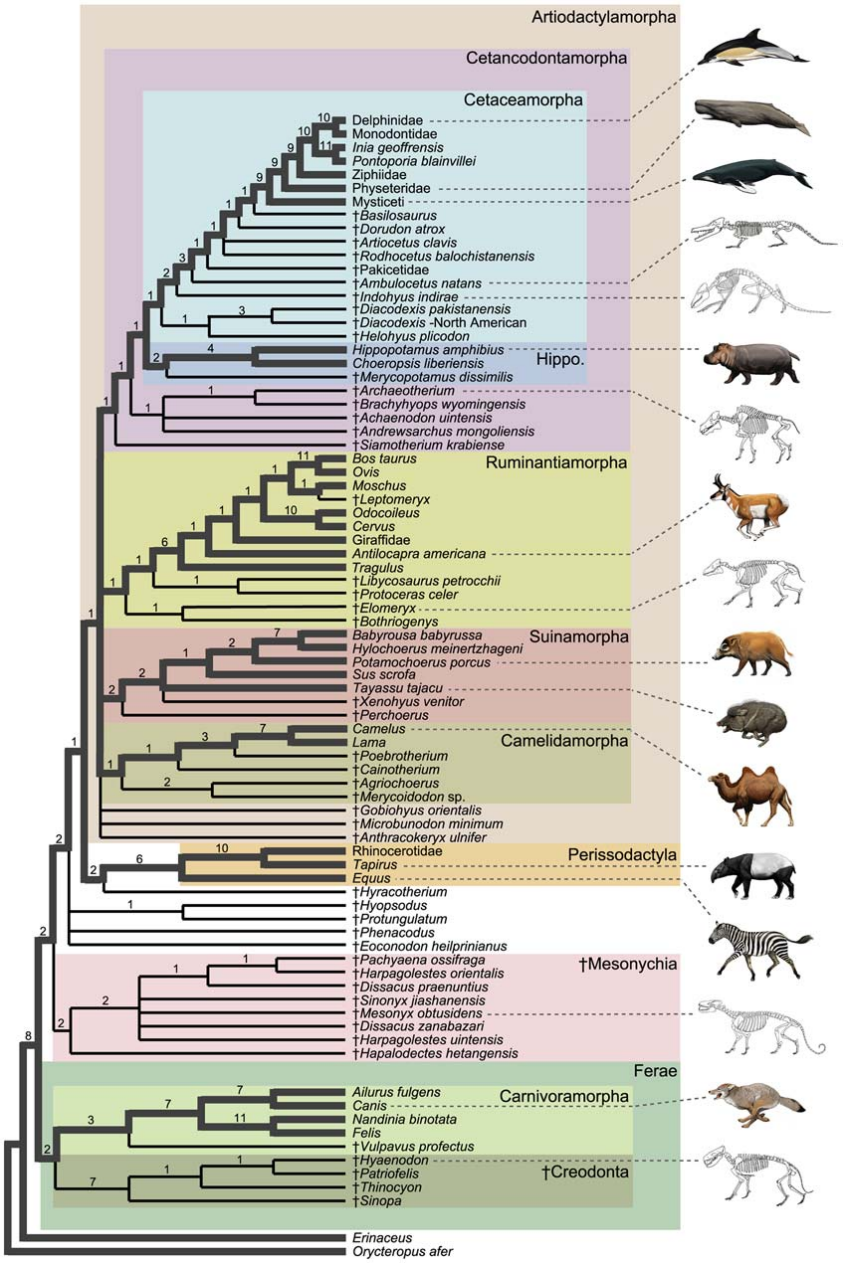
Strict consensus of 20 minimum length trees for the equally-weighted parsimony analysis of the combined data set (57,269 steps). The contents of 12 taxonomic groups, including the total clades Cetaceamorpha and Cetancodontamorpha are delimited by different colored boxes (‘Hippo’ = Hippopotamidamorpha). Lineages that connect extant taxa in the tree are represented by thick gray branches, and wholly extinct lineages are shown as thin black branches. Estimates of branch support scores are above internodes; given the complexity of the data set, these should be interpreted as maximum estimates. Illustrations are by C. Buell and L. Betti-Nash.

The total evidence matrix includes 12,222 parsimony-informative characters (603 phenotypic [osteology, dentition, soft tissue and behavior], 11,619 molecular - see Materials and Methods). Parsimony analyses in both PAUP* [36] and TNT [37] recover 20 most parsimonious trees of 57,269 steps. The strict consensus of minimum length trees is fairly well resolved (Figure 2). We rooted the trees with the tubulidentate, *Orycteropus*, as it has been found to be outside of a Carnivora + ungulate clade in a number of studies (e.g., [38], [39]). The lipotyphlan *Erinaceus* (hedgehog) is positioned at the base of the tree, followed by a split between a monophyletic Ferae and a clade of ungulates. Within Ferae, †Creodonta, Carnivoramorpha, and Carnivora are recovered. In †Creodonta, however, monophyly of the subclade †Hyaenodontidae (see [15], [17]) is not supported, as the one included †oxyaenid [15], †*Patriofelis*, nests within †Hyaenodontidae. Ferae is the sister group to a diverse clade that includes †Mesonychia, archaic ungulates of uncertain affinities, Perissodactyla and Artiodactyla. †Mesonychia is monophyletic with a basal split between the one included †hapalodectid (†*Hapalodectes*) and †Mesonychidae (†*Pachyaena*, †*Dissacus*, †*Harpagolestes*, †*Mesonyx*, †*Sinonyx*). Resolution within †Mesonychidae is limited. †Mesonychia is the sister clade to the remaining taxa in our analysis (however, see discussion below regarding the instability of this node and its effect on the overall tree). Among the remaining taxa, four archaic ungulates (†*Protungulatum*, †*Hyopsodus*, †*Phenacodus*, †*Eoconodon*) are basal to a clade composed of Perissodactyla plus Artiodactylamorpha. Within Perissodactyla, *Equus* is sister to a Rhinocerotidae plus *Tapirus* clade. †*Hyracotherium* falls outside the crown clade Perissodactyla.

Basal relationships of Artiodactylamorpha are poorly resolved in the strict consensus (Figure 2). Four major artiodactylan clades and three extinct species form a polytomy in the strict consensus. The ungrouped species are two †anthracotheriids (†*Anthracokeryx ulnifer*, †*Microbunodon minimum*) and †*Gobiohyus orientalis* (a †helohyid according to [40], and Artiodactyla *incertae cedis* according to [41]). Cetancodontamorpha, Ruminantiamorpha, Suinamorpha, and Camelidamorpha contribute to this polytomy as well.

In all of the most parsimonious trees, Camelidamorpha includes †oreodontoids (†*Merycoidodon*, †*Agriochoerus*), a †cainotheriid (†*Cainotherium*), and the extinct stem camelidamorphan †*Poebrotherium*, which is sister to the two included living camels, *Lama* and *Camelus*. Within the second major clade, Suinamorpha, †*Perchoerus* is the sister to the remaining taxa. The living tayassuid (*Tayassu*) is positioned in a polytomy with †*Xenohyus* and extant Suidae (*Sus*, *Babyrousa*, *Hylochoerus*, *Potamochoerus*). A third large clade is Ruminantiamorpha, which here includes taxa traditionally classified as ruminants, as well as three †anthracotheriids (†*Bothriogenys*, †*Libycosaurus*, †*Elomeryx*) and a †protoceratid, †*Protoceras*. In crown clade Ruminantia, *Tragulus* is sister to extant pecorans (*Antilocapra*, Giraffidae, *Cervus*, *Odocoileus*, *Ovis*, *Bos*, *Moschus*) and the extinct †leptomerycid, †*Leptomeryx*. A close relationship between the moschid (*Moschus*),†*Leptomeryx*, and Bovidae (*Bos*, *Ovis*) is supported.

Within Artiodactylamorpha, the fourth large clade recovered is Cetancodontamorpha. The †anthracotheriid †*Siamotherium* is sister to the remaining cetancodontamorphans (Figure 2). Among these, two †entelodontids (†*Brachyhyops*, †*Archaeotherium*) cluster with the †helohyid †*Achaenodon* and †*Andrewsarchus*, the latter being a relatively incomplete fossil from Mongolia that has been historically difficult to classify [42]. Within the crown group Cetancodonta, Hippopotamidae (*Hippopotamus*, *Choeropsis*) clusters with the †anthracotheriid, †*Merycopotamus*, and this clade is in turn sister to Cetaceamorpha. The basal division in Cetaceamorpha is between a clade composed of the †helohyid †*Helohyus* plus the †dichobunid genus †*Diacodexis*, and a second clade that includes †*Indohyus*, *Pakicetus*, †*Ambulocetus*, †*Rodhocetus*, †*Artiocetus*, †*Dorudon*, †*Basilosaurus*, and crown Cetacea (Mysticeti, Physeteridae, Ziphiidae, *Pontoporia*, *Inia*, Monodontidae, Delphinidae). Within this second clade, †*Indohyus* is the sister taxon of others, and there is a pectinate arrangement of extinct taxa at the base of crown Cetacea. Within crown clade Cetacea, Mysticeti is the sister group to Odontoceti. Among odontocetes, Physeteridae is basal, followed by Ziphiidae. *Pontoporia* plus *Inia* cluster, and this group is sister to a clade composed of Monodontidae and Delphinidae.

**Figure 3:**
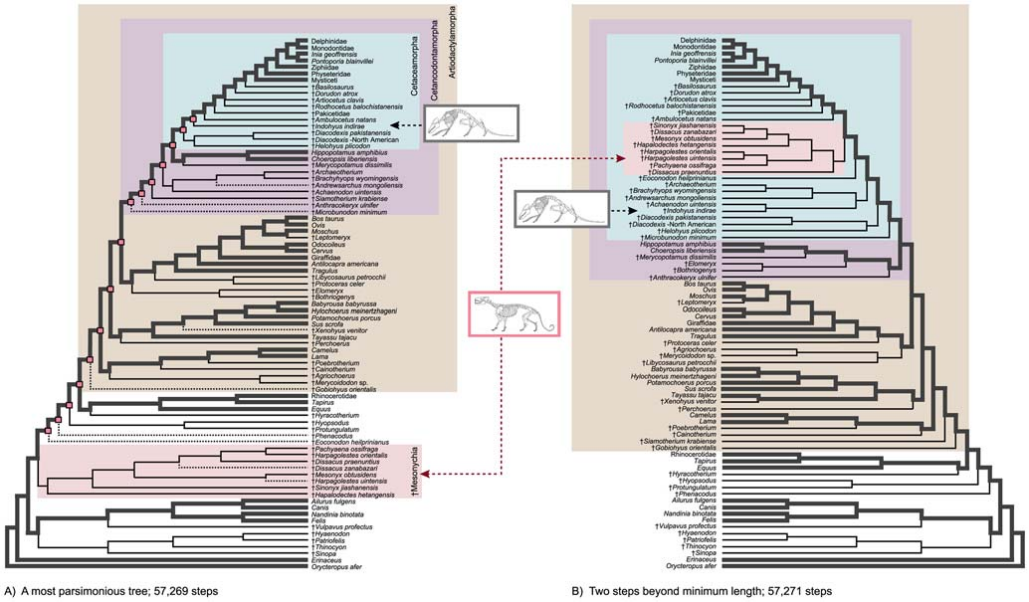
Comparison of one minimum length tree with agreement subtree superimposed (A) and a topology that is two steps beyond minimum length (B). Tree A is 57,269 steps; tree B is 57, 271 steps. Tree (A) shows one of twenty minimum length trees. Dashed branches in the minimum length topology connect to nine unstable taxa that were eliminated in the agreement subtree. Disregarding these nine taxa, relationships among the remaining 72 taxa in this tree are found in all 20 minimum length trees supported by the total evidence matrix. Tree (B) is two steps longer than minimum length. Note the highly discrepant positions of †Mesonychia and the †raoellid, †*Indohyus*, in the two trees. Small red squares at internal nodes mark clades that collapse with the movement of †Mesonychia from outside Artiodactyla (A) to within Cetaceamorpha (B). Taxonomic groups are delimited by colored boxes as in Figure 2. Illustrations are by C. Buell and L. Betti-Nash.

Unresolved sections of the strict consensus result from various equally-parsimonious placements of nine fossil taxa (Figure 3A). If the unstable positions of these taxa are ignored, relationships for the remaining 72 taxa in the analysis are consistent across all 20 minimum length trees. This “maximum agreement subtree” [43] reveals additional resolution at the base of Artiodactyla. Among the primary divisions of extant artiodactylans, Cetancodonta (Cetacea + Hippopotamidae) groups closest to Ruminantia (together, Cetruminantia) with Suina, and Camelidae branching as successively more distant relatives of Cetancodonta (Figure 3A). This basic pattern is consistent with several previous phylogenetic analyses of molecular and combined data (see discussion in [1]).

The wholly extinct †Mesonychia, a group of apparently carnivorous mammals from the Paleocene-Eocene periods (∼60-40 mya), has been implicated in the early evolutionary history of Cetacea [44]. †Mesonychians traditionally have been assigned to the stem lineage of Cetacea [8], [45], [46], [47], [48], or alternatively have been positioned completely outside of Artiodactyla [3], [5], [9], [31], [49], [50], [51]. The earliest combined phylogenetic analyses of molecules and fossils [52], [53] included information from two different morphological matrices with extensive DNA sequence data, but this work could not place †Mesonychia consistently. Equally parsimonious trees put this critical taxon deep within Artiodactyla and close to Cetacea, or completely outside of Artiodactyla and distant from Cetacea. This internal conflict resulted in a lack of resolution in strict consensus trees.

O'Leary and Gatesy [1] presented the most recent and extensive compilation of evidence bearing on whale origins, over 600 phenotypic characters and >40,000 molecular characters. In that study, the balance of evidence tipped toward a close relationship between Cetacea and †Mesonychia, with this grouping nested at least five nodes within Artiodactyla. The present study returns a phylogenetic hypothesis that contradicts O'Leary and Gatesy [1]. Instead we find minimum length trees in which †Mesonychia is the sister group to a large clade composed of Artiodactyla (including Cetacea), Perissodactyla, †*Hyopsodus*, †*Protungulatum*, †*Phenacodus*, and †*Eoconodon* (Figure 2 and 3A). The change in topology can be attributed to a more comprehensive sampling of characters and taxa in the present analysis. The critical importance of sampling is also emphasized when our results are compared with those of Thewissen et al. ([3]:Figure 1A). Despite the incorporation of critical new character data for the †raoellid †*Indohyus*, our combined analysis of molecules and morphology supports trees that are highly incongruent with the morphological analysis of Thewissen et al. [3] and more similar to those found by other authors [9], [31]. The close relationship between †*Indohyus* and Cetacea is the primary agreement among all of these analyses. The inclusion of this newly discovered †*Indohyus* material is particularly important for the impact it has on the position of †Mesonychia (see below).

It is important to note that before hypotheses supporting a close relationship between †Mesonychia and Cetacea, †mesonychians were included in †Creodonta [54]. The modern concept of †Creodonta is more restricted, excludes †Mesonychia, and is composed of two sub-clades: †Hyaenodontidae and †Oxyaenidae [15]. †Creodonts, in turn, have been grouped with Carnivoramorpha (cats, dogs, and close fossil relatives) in a more inclusive clade, Ferae [13], [16]. In our total evidence analysis †Creodonta, Carnivoramorpha and Ferae are all supported (Figure 2), and there is no support for including †Mesonychia within †Creodonta or Ferae.

Supplementary Table S1 lists synapomorphies for several key clades examined in this study. Cetacea is supported by 24 unambiguous synapomorphies, primarily from the cranium. Cetaceamorpha is also supported primarily by cranial synapomorphies. Examination of the node allying †*Indohyus* with other cetaceamorphans indicates that presence of the pachyostotic bulla is one key feature supporting this clade. The base of Cetaceamorpha is united by the presence of a third trochanter and the absence of a meatal tube on the auditory bulla. The condition of the auditory tube in basal cetaceamorphans is only recorded for †*Diacodexis pakistanensis* [55], [56] (other taxa are represented by “?” for this feature) as inferred from a line drawing in the cranial description of this specimen (the original specimen is lost to science, personal communication, J. G. M. Thewissen). This drawing suggests that the meatal tube is essentially absent, a very rare feature for noncetacean artiodactylans. It would be extremely important to corroborate this observation by discovering additional specimens of †*Diacodexis*. Hippopotamidamorpha is supported by 12 unambiguous synapomorphies from the cranium, the dentition, and the postcranial skeleton; Cetancodonta is diagnosed by 8 synapomorphies that are primarily cranial.

Regarding the outgroup taxa sampled, we recover a suite of synapomorphies for Ferae from different anatomical systems (Supplementary Table S1). Wyss and Flynn [13] previously suggested that some of these characters (carnassial shear and features of the ankle) are Ferae synapomorphies, but the majority represent new features, not previously discussed, that link †Creodonta and Carnivoramorpha. †Creodonta also is supported by a diverse set of synapomorphies, which have not previously been identified. However, five synapomorphies of †Creodonta are not found in †*Patriofelis*. If the tree is constrained for †hyaenodontid monophyly, these five characters serve as synapomorphies for the clade Hyaenodontidae, with the remaining synapomorphic characters optimizing as †creodontan synapomorphies. If the position of †*Patriofelis* is ignored, the topology for the included †hyaenodontids agrees with [17] but not Gunnell [15].

### Nodal Support and the Instability of †Mesonychia

We used three different approaches to describe the stability of our phylogenetic results: branch support [57], linked branch support [58], and selective removal of taxa and characters (see Materials and Methods). The first two methods summarize the net amount of character evidence for a particular clade or set of clades. The third assesses the phylogenetic impact of new taxa sampled here and provides insight into contrasting signals from different types of character data partitions.

Branch support scores for nodes found in the total evidence parsimony analysis range from +1 to +11 (Figure 2). Clusters of relatively high branch support generally are confined to subclades of the strict consensus that contain only extant lineages. The crown groups Cetacea, Ruminantia, Perissodactyla, and Carnivora each are characterized by at least two clades with branch support greater than +5, and Cetacea includes six groups with branch support of at least +9 (Figure 2). A grouping of living and extinct whales (†*Pakicetus*, †*Ambulocetus*, †*Rodhocetus*, †*Artiocetus*, †*Dorudon*, †*Basilosaurus*, Mysticeti, Physeteridae, Ziphiidae, *Pontoporia*, *Inia*, Monodontidae, Delphinidae) has branch support of +3, and the †raoellid †*Indohyus* is sister group to this clade with a branch support of +2. Crown group Ruminantia is well-supported (+6), as is Camelidae (+7), Hippopotamidae (+4), Carnivora (+7), the wholly-extinct †Creodonta (+7), and separation of Lipotyphyla plus *Orycteropus* from the remaining taxa (+8). Ferae (+2), †Mesonychia (+2), Artiodactyla (+1), Cetaceamorpha (+1), Hippopotamidamorpha (+2), and Cetancodontamorpha (+1) are resolved, but are not particularly robust nodes as assessed by branch support (Figure 2).

As noted above, the phylogenetic relationships of †Mesonychia have been particularly unstable in recent phylogenetic analyses. Here, †Mesonychia occupies a basal position in our most parsimonious total evidence trees, falling completely outside of a large clade that includes Artiodactylamorpha, Perissodactyla, and a variety of archaic ungulate genera. Multiple nodes separate †Mesonychia from Cetaceamorpha (Figures 2 and 3A), but examination of slightly suboptimal topologies reveals a set of trees in which †Mesonychia assumes an apical position in the tree, nested within Cetaceamorpha, Cetancodontamorpha, Cetruminantiamorpha, and Artiodactylamorpha (Figure 3B). Displacement of †Mesonychia from the base of the tree disrupts eight basal nodes supported by the total evidence, including the close relationship between the †raoellid †*Indohyus* and Cetacea (Figure 2). The sum of branch support scores for the eight nodes is +10, but the simultaneous collapse of all eight nodes in a single tree requires only two extra steps. Linked branch support for the entire set of eight clades is therefore +2. This pattern of interdependent support for adjacent nodes suggests that homoplasy is clumped and not dispersed evenly across the tree [58]. In other words, there is conflicting character support for two very different sets of alternative topologies. †Mesonychia either falls within Cetaceamorpha (Figure 3B) or is completely excluded from Artiodactyla (Figure 3A), but all other possible placements of †Mesonychia are less parsimonious than these two, highly discrepant alternatives. The pattern implies profound character conflict relating to the position of this one group, and the volatility of this critical fossil taxon limits branch support scores at multiple nodes within Artiodactylamorpha (Figure 2). Note, however, that the movement of †Mesonychia in these topologies does not affect the robustly supported relationships among extant artiodactylans in our total evidence matrix (Figure 3; thick gray branches).

**Figure 4:**
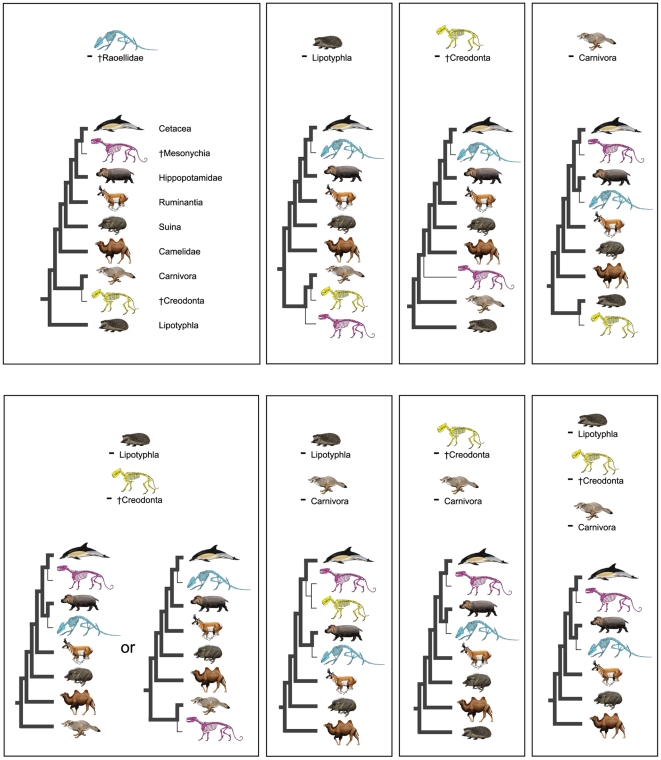
Stability of phylogenetic results to the exclusion of particular taxa from the total combined data matrix. Taxa deleted in each parsimony search are indicated above the phylogenetic result for each reanalysis. For simplicity, only the placements of major extant lineages and three critical fossil groups (†Mesonychia, †Creodonta, and †Raoellidae) are shown in the figure. Successive deletion of particular taxa from analysis results in contradictory interpretations of phylogenetic relationships. With the removal of Lipotyphla + †Creodonta, note that two equally parsimonious “islands” of trees are supported (†Mesonychia deep within Artiodactyla or completely outside the clade). †Creodonta is excluded from Artiodactyla in most reanalyses, but with the removal of Lipotyphla and Carnivora, †Creodonta clusters with †Mesonychia in a clade that is the sister group to Cetacea. Illustrations are by C. Buell and L. Betti-Nash.

The instability of †Mesonychia also is apparent from parsimony analyses in which taxonomic sampling is perturbed. Carnivora, †Creodonta, and Lipotyphla (*Erinaceus*) were removed successively from the total evidence matrix, in a variety of combinations, and parsimony searches re-run (Figure 4). Whenever Carnivora is deleted, †Mesonychia groups close to Cetacea and †*Indohyus* clusters with Hippopotamidae in contrast to the total evidence result (Figure 2). For the analysis in which both Carnivora and Lipotyphla are extracted, †Creodonta groups with †Mesonychia as the sister group to Cetacea (Figure 4). Removal of either Lipotyphyla or †Creodonta alone does not result in a repositioning of †Mesonychia within Artiodactyla, but deletion of both Lipotyphla and †Creodonta gives an ambiguous answer. There are two equally parsimonious sets of very different trees; one set positions the †raoellid †*Indohyus* close to Cetacea with †Mesonychia completely excluded from Artiodactyla, while the other set joins †Mesonychia with Cetacea in a clade that is deeply nested within Artiodactyla (Figure 4). This pattern mirrors that seen in examination of nearly optimal trees (Figure 3). Overall, the phylogenetic placements of †Mesonychia and †*Indohyus* are highly sensitive to the particular outgroup taxa included in analysis. Representatives from Carnivora, †Creodonta, and Lipotyphla are all required to give clarity to character polarities. If only one of these groups is present, the positions of †Mesonychia and †*Indohyus* changes dramatically. This underscores not only the importance of broad taxon sampling, but also the instability of †Mesonychia and †*Indohyus*, particularly when compared to the relatively stable phylogenetic ‘backbone’ of Artiodactyla that is supported by data from extant lineages.

Excluding the controversial postcranial evidence for †*Indohyus* has little effect on phylogenetic results; nine optimal trees were recovered that were a subset of the 20 minimum length trees for the total evidence matrix. The strict consensus of these nine trees is slightly more resolved than the strict consensus derived from the complete combined data set (Figure 2). Removal of †*Indohyus* entirely has a much more profound effect. †Mesonychia again moves from a basal position to a highly nested placement within Artiodactyla, close to Cetacea (Figure 4). This suggests that the unique combination of characters in the skull of †*Indohyus* has a very large influence in determining results in combined analysis of molecules and morphology.

**Figure 5:**
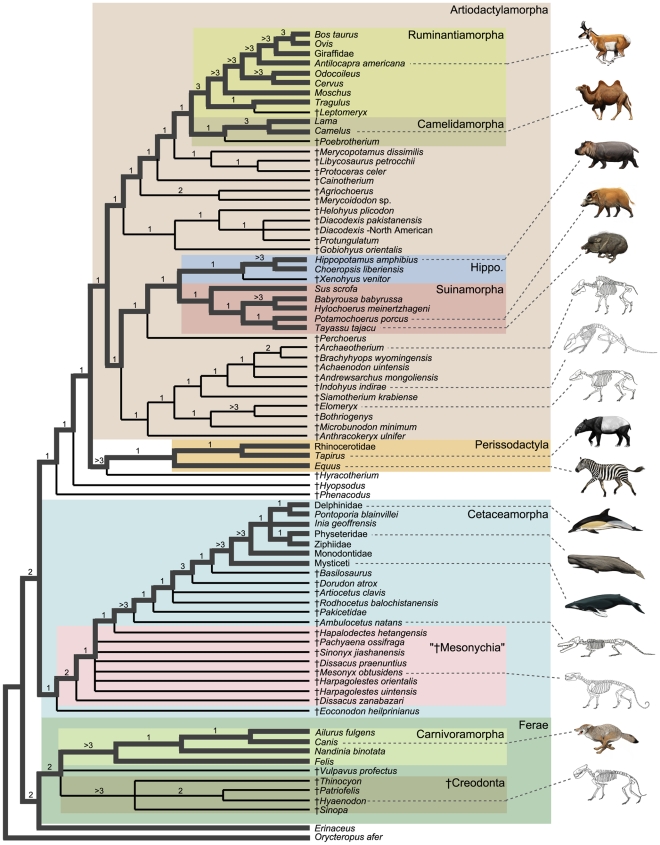
Strict consensus of the 48 minimum length trees for the equally-weighted parsimony analysis of 606 characters observable in fossils (3,722 steps). Note that both Selenodontia (Ruminantia + Camelidae) and Suiformes (Hippopotamidae + Suina) are supported, in contrast to the total evidence analysis (Figure 2). Colored boxes that delimit taxonomic groups are as in Figure 2 (Hippo. = Hippopotamidamorpha).

Additional perturbations of the total evidence matrix included analysis of only skeletal and dental characters (characters that fossilize), and a search that considers only molecular, soft tissue, and behavioral characters (those for which we generally lack data for the fossils sampled). Analysis of molecular characters, soft anatomy, and behavior from extant taxa yields a tree that is generally consistent with the total evidence analysis (Figure 2). Separate analysis of characters that commonly fossilize supports a very different result (Figure 5). The traditional artiodactylan clades Selenodontia (Ruminantia + Camelidae) and Suiformes (Hippopotamidae + Suina) are supported, and †*Indohyus* does not group close to Cetacea. Instead, Cetacea clusters within a paraphyletic †Mesonychia, and this grouping is excluded from crown clade Artiodactyla. This means that the recent discovery of fossils such as †*Indohyus* [3] and †*Rodhocetus* [4], which are critical taxa in early cetaceamorphan evolution did not result in congruence between phylogenetic data from the fossil record and data from living taxa (see also discussion in [1]). The skeletal and dental characters alone do support Ferae, Carnivora, Caniformia, and †Creodonta [Figure 5]), but overall, there is extensive conflict with the total evidence analysis. If the combined data matrix is fit to the topologies generated by the skeletal+dental data, these trees are at the least 1,500 steps beyond the minimum length.

### Selected Character Optimizations for Cetancodonta

#### Character reconstructions based on parsimony alone

Since the recognition that whales are highly-derived artiodactylans, it has been of interest to understand how an aquatic, carnivorous clade, Cetacea, evolved within a predominantly terrestrial, herbivorous clade, Artiodactyla. Our primary means of reconstructing characters in hypothetical common ancestors, and of reconstructing soft tissue/behavior characters (that are not directly observable) in fossil taxa, is to use parsimony [59], [60]. We have coded the following behavioral characters in our matrix: aquatic habitat (character 618 [state 1]), herbivorous diet (character 658 [state 2]), and ability to interpret the direction of sounds under water (character 659 [state 1]). These three character states optimize unambiguously to the common ancestor of Cetancodonta.

This corroborates predictions about the origin of an aquatic lifestyle as having occurred once in the common ancestor of Hippopotamidae and Cetacea [27]. This common ancestor of Cetancodonta had the derived behavior of spending at least 10% of its time in water (character 618 [state 1]). Parsimony indicates that this state is shared by all extant members of Cetancodonta, and is reconstructed for all taxa nested within Cetancodonta (including basal cetaceamorphans, such as †*Indohyus*, †*Diacodexis* and †*Helohyus*). Gatesy et al. [27] had previously suggested overturning a parsimony-based optimization for some extinct taxa nested in this clade based on absence of osteological correlates for aquatic behavior, but we do not advocate that position here (also see [1], [2]).

Using parsimony we reconstruct all fossil hippopotamidamorphans as herbivorous (character 658 [state 2]). The dietary behaviors of taxa along the stem to Cetacea (i.e., noncetacean cetaceamorphs) are, however, equivocal based on optimization of states seen in extant taxa. Somewhere on the stem to Cetacea, diet changed from herbivory to aquatic carnivory (character 658 [state 3]), but using parsimony alone we cannot reconstruct unambiguously where the behavioral change occured Parsimony-based optimization also implies that all living and extinct cetancodontans shared a derived ability to hear underwater sounds at least as well as extant *Hippopotamus*. This is noteworthy because several cetancodontans (including living members of Hippopotamidae) lack a pachyostotic auditory bulla (involucrum) in the ear.

To summarize, in the minimum length trees (e.g., Figure 3A), the †raoellid †*Indohyus* is reconstructed to have spent at least 10% of its time in water and to have had the derived behavior of directional underwater hearing, but reconstruction of its diet is equivocal. In alternate trees that are two steps longer, parsimony recovers the same character state reconstructions for †Mesonychia, because this taxon is a close relative of Cetacea in slightly longer trees (e.g., Figure 3B).

### Extended character reconstructions: inferring behavior from osteology and dentition

As discussed by [33], [60] inferences about behavior in fossil taxa, which go beyond parsimony, can be made if there is “compelling morphological evidence” that a certain fossilized trait is strictly correlated with a certain behavior (e.g., distinctive coiling of the cochlea in bats indicating echolocation [61]). These deductions should, however, be clearly delineated from reconstructions based on parsimony. Here we discuss such inferences related to diet and hearing in Artiodactyla.

Molars that have a tall, angular protoconid and a compressed talonid are typically associated with carnivorous diets in mammals, and molars with low-crowned, quadritubercular cusps are associated with herbivory/omnivory [6], [7]. Reconstructing behavior from fossilized tooth shape, we would infer that several cetaceamorphans (†*Diacodexis*, †*Helohyus*, and †*Indohyus*) are herbivorous/omnivorous because they have quadritubercular teeth (hypocone on M2, character 419 [1]). It is noteworthy that prior to the description of a relatively complete †*Indohyus* skull there were no cetaceamorphans that had both the pachyostotic ear region and quadritubercular dentition.

Molar shape would suggest that cetaceamorphans from †*Ambulocetus* through more highly nested taxa were carnivorous due to the presence of narrow talonids on m2 (character 364, state 1) and of tall protoconids on m1 (358, state 1; here technically †*Pakicetus* and more highly nested taxa due to missing data). According to minimum length trees for the combined data, these character states were independently derived within Cetaceamorpha and in †Mesonychia (Figure 3A). In the slightly longer topology (Figure 3B), however, both of these dental characters are synapomorphies uniting †Mesonychia and Cetacea. Based on the results of the total evidence analysis, we can return to the question posed in the introduction, ‘Did aquatic carnivory precede committed life in the water?’ Inferring carnivory from tooth shape and inferring committed life in the water from detachment of the sacral vertebrae from the pelvis (character 488, state 0), carnivory did precede committed life in the water. Not until the last common ancestor of †*Dorudon*, †*Basilosaurus*, and crown Cetacea did cetaceamorphans lose the articulation between the pelvis and the vertebral column, but dentition suggesting carnivory appears at a more basal node (see also [62]).

The presence of the pachyostotic bulla and what it implies about hearing has also been of interest because this is a relatively rare anatomical feature among mammals. This structure has been argued to indicate an ear region derived to process underwater sounds [8], a behavior that has evolved in Cetaceamorpha. Luo and Gingerich ([8]:89) stated that acoustic isolation of the left and right ears creates density differences analogous to those created by a pachyostotic bulla, and may confer directional hearing underwater. Interestingly, *Hippopotamus* lacks a pachyostotic bulla despite the fact that this species has an ability to hear underwater sounds exceeding that of typical terrestrial mammals [63]. Pachyostosis of the ear region, therefore, does not appear to be essential for certain derived types of underwater hearing. Pachyostosis may indicate instead an even more derived level of underwater hearing than previously recognized but confirmation requires further functional studies.

Reconstructing auditory function from osteology, we would infer that the presence of a pachyostotic bulla (character 59, state 1) in †*Indohyus*, and all more highly nested cetaceamorphans (Figure 3A), potentially indicates an even more derived state of underwater hearing than that which developed in the common ancestor of Cetancodonta. If the alternate topology only 2 steps longer (Figure 3B) obtains in future studies, (with †Mesonychia closer to Cetacea and †*Indohyus* a more distantly related cetaceamorphan), then the pachyostotic bulla developed two times independently in Cetaceamorpha: once in the common ancestor of †*Ambulocetus* and Cetacea, and once in †*Indohyus*. Furthermore, optimization by parsimony indicates that underwater hearing is a feature shared by all cetancodontans, thus this character state appeared at a more basal node than detachment of the sacral vertebrae from the pelvis and committed life in the water (character 488, state 0).

In summary, these inferences imply that the history of Cetaceamorpha included both carnivorous and herbivorous species. All cetancodontans were at least as aquatic as living hippos and exhibited some ability to hear underwater sounds, even though several cetancodontan species lack a pachyostotic bulla. Appearance of the pachyostotic bulla may indicate a shift to a yet more derived degree of directional underwater hearing, a hypothesis that requires further investigation. In slightly longer trees, in which †Mesonychia groups close to Cetacea, the derived bulla of †*Indohyus* would be interpreted as convergent with that of cetaceans. Finally the shift to carnivory within Cetaceamorpha preceded the loss of limbs that functioned in terrestrial locomotion in this clade.

### Conclusions

This study highlights the importance of expanded taxon sampling when examining complex questions of relationships and underlying patterns of character evolution. The addition of carnivorans and †creodonts, taxa not traditionally included in discussions of artiodactylan/cetacean phylogeny, has a significant impact on the resultant tree topology (Figure 2). The complex combined data set compiled for the present study underscores some of the key issues remaining in studies of cetacean origins. Future analyses should continue to expand taxon sampling. The current matrix, although relatively large, remains highly unstable to slight perturbations in taxon sampling (Figure 4). Initiatives underway, such as the mammalian component of the Assembling the Tree of Life project [64], seek to increase both the number of taxa and characters utilized in combined data phylogenetic analyses. Such comprehensive studies will more fully explore the influence of outgroups on deeply nested ingroup relationships.

†Mesonychia is only distantly related to Artiodactyla in our shortest trees, with †*Indohyus* grouping as a close relative to living cetaceans. However, in trees just two steps longer than minimum length, we find the more ‘traditional’ arrangement of †Mesonychia positioned close to Cetacea. In these trees, †*Indohyus* is a cetaceamorphan but is not as closely related to Cetacea as is †Mesonychia. The lack of abundant support for either topology and the outstanding incongruence between data that fossilize and those that do not, suggests that many key fossils remain to be discovered.

Ferae and †Creodonta are both solidly supported clades in our total evidence analysis, each with multiple synapomorphies. This study is the largest test of the relationships of these taxa to date and utilized many characters that had never before been applied to members of the Ferae in a cladistic context. Additional work is needed to test the monophyly of these groups, such as the addition of several taxa suggested in the past to be closely related to †Creodonta (e.g., †Leptictidae) as well as much more comprehensive sampling of the †creodonts themselves. However, despite the need for further research, this analysis shows that discounting Ferae and †Creodonta as monophyletic groups [15], [17] is premature.

## Materials and Methods

### Taxon and Character Sampling

The large morphological character matrix previously compiled by O'Leary and Gatesy [1] included 71 taxa (28 extant, 43 extinct) and 635 characters (310 cranial osteology, 147 dental, 123 postcranial osteology, and 55 soft-tissue/behavior). This data set for Artiodactyla and close relatives was used as a starting point for the present analysis.

We generally chose representatives of extinct groups based on the relative completeness of fossil material. Four members of the extinct order †Creodonta were included: three †hyaenodontids (†*Thinocyon*, †*Sinopa*, †*Hyaenodon*) and one †oxyaenid (†*Patriofelis*). Five carnivoramorphans were added: one basal extinct taxon (†*Vulpavus*), and within Carnivora two extant feliforms (*Nandinia* and *Felis*), and two extant caniforms (*Canis* and *Ailurus*). We also sampled an extant lipotyphlan insectivore (*Erinaceus*). We based morphological character codings for all extant genera on examinations of single species, but when compiling DNA sequences (see below), monophyly of extant genera was assumed to reduce the overall percentage of empty cells for molecular characters in the matrix. To limit missing data for the five fossil taxa, observations from multiple species were merged for each extinct genus. All characters were scored based upon direct examination of specimens. Morphological character codings were collected and archived using the web application Morphobank [65].

A primary objective of this study was to assess the phylogenetic impact of newly described †raoellid artiodactylan fossils [3] in the context of a very large combined matrix of molecular and morphological characters [1]. In addition to the ten taxa added to the matrix, we augmented our previous set of character state observations for †*Indohyus*. M. O'Leary examined †*Indohyus* specimens in the collection of H. Thewissen at Northeastern Ohio Universities College of Medicine (NEOUCOM). We were permitted only to corroborate matrix scores for the 196 characters published in [3] because the †raoellid fossils are still being examined by Thewissen and colleagues. Thus there are missing data for this taxon that could have been collected if we were allowed to make new observations for our full matrix of 661 morphological characters.

We increased morphological character sampling slightly relative to the analysis of O'Leary and Gatesy [1]. Approximately five morphological characters were added based upon previous systematic work on Ferae [18]. This count is not exact because many characters were at first appended to the previously published matrix, but then later subsumed into existing characters once overlaps in character states were identified. Delimitations of some characters in the matrix from [18] were revised based upon new information from Ferae/Lipotyphla. There is an overall increase of 26 morphological characters relative to our previous matrix due to the addition of characters and re-defining of previous characters.

We also augmented the extensive molecular data (40,928 characters) compiled by [1]. First, data for the five new extant genera sampled here (*Canis*, *Felis*, *Nandinia*, *Ailurus*, *Erinaceus*) were added to our previously published alignments; four mitochondrial genomes and information from 31 nuclear loci at the Genbank database were added to the overall matrix. We also included additional new data from Genbank that have been published since [1]; for example, sequences from the nuclear genes *TBX4* and *SRY* were concatenated to the existing molecular data set. Finally, 49 new sequences from five nuclear genes (*ZP3*, *BDNF*, *ATP7A, AMEL, RNASE1*) were generated in our lab for this study (Genbanks #s GQ487580-GQ487628) using PCR, cloning, and sequencing methods described in [1], [27]. PCR/sequencing primers for the *ZP3* gene were (5′ to 3′): ZP3L1 - GACCAACTAAACAAAGCCTG, ZP3L2 - CAGCAAGTCCTCCAACAGGT, ZP3ODOL1–GAGACCAGATTGGACATAAC, ZP3ODOR1–GCACACAGGGTGGGAAGCAG, and ZP3R3–TATTGGGAAGCAGACAC. Published primers were used for the *ATP7A*, *BDNF*, *AMEL*, and *RNASE1* genes [66], [67], [68]. Recently deposited data in Genbank and sequences from our lab generally were aligned to our previously published matrix with the introduction of very few new gaps [e.g., see 1]. However, several gene segments were re-aligned using CLUSTALW [69] with gap opening cost of five and gap extension cost of 1; some adjacent gaps in the resulting multiple-sequence alignments were consolidated using SeqApp 1.9a [70] as in [1], [27]. All newly-incorporated loci (*TBX4*, *SRY*, *ZP3*) also were aligned in this way. The final molecular data set exceeded that of O'Leary and Gatesy [1] by more than 5,500 aligned nucleotides. The 661 morphological characters were downloaded from Morphobank and merged with the revised molecular matrix of 46,587 characters in PAUP* 4.0b10 [36]. The total combined data set for this study has been stored at Morphobank (project #48). The main matrix in this project file is the morphology component of this study, and the total evidence nexus file is in the documents folder for this project. The nexus file records all Genbank numbers for molecular sequences in the matrix. This nexus file is also available as supporting information for this article: Appendix S1.

### Phylogenetic Analyses

Parsimony analyses of the total evidence data set were undertaken using both PAUP* 4.0b10 [36] and TNT [37]. In PAUP*, searches were heuristic with 1000 random stepwise additional replicates and tree-bisection-reconnection (TBR) branch swapping. All character state changes were given equal weight, all characters were unordered, gaps were treated as missing data, and the amb- option was used so that internal branches were collapsed if minimum length was zero. The search strategy employed in TNT was to first analyze the data under the ‘New Technology search’ option, selecting the sectorial search, rachet, and tree fusing search methods, all with default parameters. Under this setting, iterations were run until the minimum length tree was found in 500 separate replicates, to try to hit as many islands of trees as possible [71]. The generated trees were then analyzed under traditional search options (using TBR) in order to more fully explore the discovered tree islands. Strict consensus trees and agreement subtrees [43] were used to examine topological conflicts among multiple most parsimonious trees.

Branch support [57] was used as a measure of nodal stability for all groups resolved in the strict consensus of minimum length trees, and linked branch support [58] was estimated to summarize the interdependence of character support for multiple nodes supported by the total evidence. Branch support for a particular clade can be defined as the length of the shortest tree that does not include the clade, minus the length of the shortest tree that includes that clade. Estimates of branch support were derived from additional heuristic searches in PAUP* that incorporated “anti-constraints,” and also by searches in TNT that retained sub-optimal trees and determined at what length each recovered clade was lost in a strict consensus. Linked branch support for a particular set of supported clades can be defined as the length of the shortest tree that does not include *any* of those clades, minus the length of the shortest tree that includes *all* of those clades [58]. Further PAUP* searches with “anti-constraints” enforced were used to estimate linked branch support for particular groups of nodes. Given extensive blocks of missing data in the combined matrix, character resampling (bootstrap) was not utilized to assess nodal support in this study, and estimates of branch support in our trees may be lower than the estimates calculated here.

The total evidence analysis of all data was considered the best test of phylogenetic relationships, but we explored other search strategies to examine different signals in the combined matrix, and to test the overall stability of our results. A variety of searches were conducted, with different subsets of taxa and characters activated in each analysis. These were: 1) All characters and all taxa included. 2) Only skeletal and dental characters for all taxa; this search summarized the strongest hierarchical signal in traits that commonly fossilize. 3) Only molecular, behavioral, and “soft anatomical” characters for extant taxa; this run shows the pattern supported by information that generally can only be coded from extant taxa. 4) All taxa and most characters, with postcranial characters from †*Indohyus* excluded. This analysis assessed whether or not our total evidence results are dependent on possible misassignment of bones [11] to this critical taxon. 5) All taxa included, except for †*Indohyus*; this analysis determined the influence of this critical ‘intermediate’ taxon on phylogenetic results. 6) All characters and most taxa, with some outgroup taxa deleted from analysis. All combinations of three higher-level groups (†Creodonta, Carnivoramorpha, Lipotyphyla) were successively deleted to see the effects of outgroup sampling on phylogenetic results, in particular placement of Cetacea relative to †Mesonychia.

Characters were optimized onto all minimum length trees using the map characters option in TNT [37]. For critical nodes supported by the total evidence, character state changes that mapped unequivocally onto all optimal trees were noted; these are listed in Supplementary Table S1. We also used parsimony to map characters onto suboptimal hypotheses to identify transformations that support conflicting relationships regarding †Mesonychia and †*Indohyus*.

## Supporting Information

Table S1 Unambiguously optimized synapomorphies for selected clades (Figure 2). Symbols are *, which indicates that a character state reverses in the clade and thus is not shared by all members, and #, which indicates a contradictory state found in the ‡oxyaenid ‡Patriofelis.
(0.12 MB DOC)

Appendix S1 Matrix as a Nexus file. Matrix with both morphological and molecular information. Genbank numbers of sequences are included in the file.
(4.06 MB TXT)
