# Supplementary material for: Relationships of Cetacea (Artiodactyla) Among Mammals: Increased Taxon Sampling Alters Interpretations of Key Fossils and Character Evolution
Source: PLoS One. 2009 Sep 23;4(9):e7062. doi: 10.1371/journal.pone.0007062 (PMC2740860; doi:10.1371/journal.pone.0007062)
Supplement: Table S1 — Unambiguously optimized synapomorphies for selected clades (Figure 2). Symbols are *, which indicates that a character state reverses in the clade and thus is not shared by all members, and #, which indicates a contradictory state found in the ‡oxyaenid ‡Patriofelis. (0.12 MB DOC) [file pone.0007062.s001.doc]

Supplementary Table 1 - Unambiguously optimized synapomorphies for selected clades (Figure 2).

| Cetacea |  |
| --- | --- |
|  | 7: Prefacial commissure fossa of the petrosal: absent (convex-flat or a bump) (0) |
|  | 29: Caudal tympanic process shape: mediolaterally narrow, bar of bone medial to stapedial muscle fossa (0)* |
|  | 49: Mastoid region external exposure on posterolateral face of braincase: not exposed (1 |
|  | 145: Hard palate lateral aspect forms U-shape at posterior extreme: absent (1) |
|  | 161: Vomer exposure between maxillae on palate: present (0)* |
|  | 192: Thickness of zygomatic arch: splint (1) |
|  | 198: Shape of lacrimal facial process: greater height than length (2) |
|  | 199: Posterior margin of external nares: distal to all teeth or baleen (3) |
|  | 204: Facial crest: angled, well-developed facial tubercle (1) |
|  | 205: Nasal relative to premaxilla anterior extreme: retracted strongly (2) |
|  | 208: Crests for temporalis muscle: separate (0) |
|  | 224: Position of maxillary (infraorbital) foramina: superior to orbit (1) |
|  | 237: Position of anterior margin of orbit relative to particular teeth: posterior to teeth or baleen (4) |
|  | 255: Coronoid process superior margin: inferior to condyle (1) |
|  | 288: Postorbital constriction: absent (1) |
|  | 295: Premaxillae: contribute to vertex of skull (1)* |
|  | 306: Mesorostral canal: present (1) |
|  | 314: Telescoping: present (1) |
|  | 317: Nasal aperture location: posterior of rostrum (1) |
|  | 318: Mesethmoid exposure: exposed dorsally (1)* |
|  | 320: Nasal passage orientation: vertical (0) |
|  | 407: Teeth in premaxilla: absent (1) |
|  | 480: C6 with largest cervical transverse processes: absent (0)* |
|  | 525: Independent trapezium: absent (1) |
|  |  |
| ✝*Indohyus* + Cetacea |  |
|  | 59: Involucrum of bulla: present (1) |
|  | 104: Size of falcate process: large (1)* |
|  | 559: Astragalar neck: long (1)* |
|  |  |
| ✝*Diacodexis* + ✝*Helohyus* |  |
|  | 20: Transpromontorial sulcus: present (0) |
|  | 103: Falcate processes: absent (1) |
|  | 124: Mediolateral dimensions of the squamosal portion of external acoustic meatus: half of the mediolateral dimensions of the petrosal or less (0)* |
|  | 356: m1 paraconid: present (0) |
|  | 381: Lower molar m2 metaconid position: slightly distal (2) |
|  | 425: P4 paracone relative to M1 paracone: equal/subequal to height of paracone of M1 (0) |
|  |  |
| Cetaceamorpha |  |
|  | 62: Auditory bulla internal structure: hollow (1) |
|  | 71: Meatal tube of tympanic: absent (0) |
|  | 94: Composition of anterior wall of sulcus: formed by mastoid process of petrosal (0) |
|  | 95: Mastoid region exposed in ventral view: present (1)* |
|  | 152: Tip of palate: narrow, medio-lateral width less than distance between posterior most teeth (1) |
|  | 230: Lacrimal tubercle: absent (0)* |
|  | 234: Jugal contribution to postorbital bar: mostly frontal (2) |
|  | 278: Caudal nasal separated by anterior incursion of frontal: present (1) |
|  | 322: Lower canine size: same size as incisors (1) |
|  | 342: p3 shape: no talonid (1)* |
|  | 540: Third trochanter of femur: present (0)* |
|  | 545: Type of tibia-fibula fusion: fused proximally and distally (1) |
|  | 605: Distal phalanges in dorsal view: phalanx compressed transversely (0) |
|  |  |
| Hippopotamidamorpha |  |
|  | 98: Separate, enclosed, foramen ovale: absent (1) |
|  | 163: Buried palatine grooves: present (1) |
|  | 180: Knob superior to foramen magnum: absent (0) |
|  | 242: Contact of maxilla with frontal: absent (1) |
|  | 248: Angular process lateral expansion: present (1) |
|  | 331: Lower canine shape in cross section: triangular (1) |
|  | 350: p4 metaconid: present (1) |
|  | 448: Paraconule on m2: absent (0) |
|  | 497: Distal humerus, medial border of trochlea: slanted (0) |
|  | 542: Patellar groove symmetry: medial asymmetry (0) |
|  | 556: Astragalus; shape of major part of sustentacular facet: subtle division into two M-L planes both with convexity (1) |
|  | 562: Anterior digital fossa: present (1) |
|  |  |
| Cetancodonta |  |
|  | 2: Subarcuate fossa: absent (1) |
|  | 7: Prefacial commissure fossa of the petrosal: present concave (1)* |
|  | 8: Shape of tegmen tympani: pronounced convexity (1)* |
|  | 10: Degree of inflation of the tegmen tympani: hyperinflation (0)* |
|  | 14: Vascular groove on the lateral surface of the tegmen tympani: present (0)* |
|  | 275: Shape of each nasal at the anteromesial tip: blunt (0)* |
|  | 400: Diastemata between incisors: present (1) |
|  | 422: P4 postprotocrista: absent (0)* |
|  |  |
| Cetancodontamorpha |  |
|  | 425: P4 protocone relative to M1 paracone: greater than twice the height of M1 paracone (1)* |
|  | 431: M1 parastyle: absent (0)* |
|  |  |
| Artiodactylamorpha |  |
|  | 557: Sustentacular facet width: wide (1)* |
|  | 569: Contact of distal astragalus with cuboid: present (0) |
|  | 583: Ectal facet primary orientation: lateral (1) |
|  | 587: Astragalus lateral edges of proximal and distal trochlea: aligned (1)* |
|  | 588: Astragalar head arc – dorsoplantar: wide arc ~ 200 degrees (1) |
|  | 589: Astragalar neck width relative to tibial trochlea: neck as wide as tibial trochlea (1)* |
|  |  |
| Ferae |  |
|  | 29: Caudal tympanic process shape: mediolaterally narrow, bar of bone medial to stapedial muscle fossa (0) |
|  | 51: Median furrow: absent (1) |
|  | 178: Mastoid foramen: absent (0)* |
|  | 430: Carnassial shear anywhere on toothrow: present (1) |
|  | 447: Stylar shelf on M2: present (0)* |
|  | 455: M2 preparacrista orientation: mesiobuccal (1)* |
|  | 555: Angle between lateral calcaneus and sustentaculum: 180 degrees (0) |
|  | 559: Astragalar neck: long (1)* |
|  | 600: 1st metatarsal length: greater than/equal to 50% length of 3rd metatarsal (0)* |
|  | 604: Ventral border of distal phalanges: curved inferiorly (0) |
|  |  |
| ✝Creodonta |  |
|  | 110: Medial glenoid pit: present (1) |
|  | 128: (Bulla off) Jugular (posterior lacerate) foramen and basicapsular fissure: presence of confluence, well-defined (1)# |
|  | 160: Palatine foramen location: contained entirely within premaxilla (0)# |
|  | 182: Paracondylar (paramastoid or paroccipital) process length: long, extends inferior to inferior margin of bulla (0) |
|  | 193: Lacrimal fossa (an antorbital pit in lacrimal): present (1) |
|  | 225: Maxillary (infraorbital) foramen: position: at P3 or anterior (3) |
|  | 229: Midline frontal depression: present (1)# |
|  | 273: Posterior frontal border: straight (1)# |
|  | 277: Lateral margins of the nasals at the caudal extreme: divergent then convergent (3) |
|  | 329: Position of canine relative to incisors (lower): canine lateral or anterior (1) |
|  | 369: m2 talonid morphology of hypoconulid: due to large hypoconulid, tooth has elongate oval outline (0) |
|  | 412: Number of roots on P1: two/three (1) |
|  | 433: M1 metastyle shelf: projects farther labially than parastyle shelf (0) |
|  | 460: M2 postmetacrista orientation: distobuccal (1) |
|  | 463: M2 parastyle: crest (1) |
|  | 541: Patellar articular surface on femur: narrow (1) |
|  | 557: Sustentacular facet width: Narrow, < or = to 50% width of the astragalus (0) |
|  | 569: Contact of distal astragalus with cuboid: present (0) |
|  | 576: Ectal facet (lateral process) of the astragalus - shape regardless of orientation: flat-gently concave (1)* |
|  | 586: Lateral side of sustentacular facet: bent onto lateral side of astragalus to articulate with calcaneus (1)# |

Symbols are *, which indicates that a character state reverses in the clade and thus is not shared by all members, and #, which indicates a contradictory state found in the ✝oxyaenid ✝*Patriofelis.*
